# Posttraumatic Stress Symptoms, Buffering Resources, and Avoidant Coping Strategies in Patients With Cirrhosis: A Latent Class Analysis

**DOI:** 10.1002/hsr2.73319

**Published:** 2026-11-05

**Authors:** Jintao Cheng, Huiqiong Gui, Suyun Guo, Yihua Yang, Yun Ran

**Affiliations:** ^1^ Liver Disease Department Beijing University of Chinese Medicine Shenzhen Hospital (Longgang) Shenzhen Guangdong China; ^2^ Department of Nursing Beijing University of Chinese Medicine Shenzhen Hospital (Longgang) Shenzhen Guangdong China

**Keywords:** avoidant coping, latent class analysis, liver cirrhosis, posttraumatic stress symptoms, sense of control, social support

## Abstract

**Objective:**

To identify latent classes of posttraumatic stress symptoms (PTSS), buffering resources (social support and sense of control), and avoidant coping strategies among patients with cirrhosis, and to examine demographic and clinical factors associated with class membership.

**Methods:**

From October 2024 to May 2025, patients with cirrhosis were recruited by convenience sampling from a tertiary hospital in Shenzhen, Guangdong Province. Participants completed the Perceived Social Support Scale (PSSS), PTSD Checklist‐Civilian Version (PCL‐C), Spheres of Control Scale (SCS), and Self‐Report Coping Scale (SRCS). Latent class analysis was used to identify subgroups, followed by multinomial logistic regression to examine associated factors.

**Results:**

A total of 316 patients were included. Mean scores were 60.78 ± 16.00 for social support, 30.67 ± 11.99 for PTSS, 48.42 ± 12.07 for sense of control, and 29.53 ± 9.26 for avoidant coping. Three latent classes were identified: the Buffering Type—Low Avoidance/Symptoms Group (64.2%), the Buffering Deficiency— Symptoms/Avoidance Dilemma Group (7.5%), and the Avoidant Coping—Symptoms Dilemma Group (28.2%). In the pooled adjusted model, decompensated cirrhosis differentiated Class 2 from Class 1, while unmarried status, monthly income < 5000 RMB, unemployment, and decompensated cirrhosis differentiated Class 3 from Class 1. Although gender differed across classes in univariate analysis, the adjusted C1‐versus‐C3 gender effect was not statistically significant (OR = 0.780, 95% CI 0.365–1.667, *p* = 0.523). Because Class 2 contained only 24 participants, estimates involving this class should be interpreted cautiously.

**Conclusion:**

Patients with cirrhosis showed heterogeneous profiles of PTSS, buffering resources, and avoidant coping. Disease stage and selected social and economic characteristics were associated with class membership. The small Class 2 should be considered a provisional profile requiring replication before firm class‐specific clinical conclusions are drawn.

## 1 Introduction

Liver cirrhosis is a progressive chronic liver disease characterized histopathologically by diffuse hepatocyte necrosis, abnormal hepatocyte regeneration, angiogenesis, extensive fibrous tissue hyperplasia, and pseudolobule formation [1]. Existing data show that in 2017, there were approximately 112 million cases of compensated cirrhosis and about 10.6 million cases of decompensated cirrhosis globally, with approximately 1.32 million patient deaths due to cirrhosis [2]. From 2002 to 2010, the mortality rate of cirrhosis patients in South Korea was about 30.66%, slightly higher than that of the five major cancers [3]. China has about 300 million patients with liver diseases, accounting for approximately 11% of global deaths [2, 4]. The disease has a long course, low cure rate, numerous complications, and slow progression, causing severe harm to patients [5]. The compensated stage has no obvious clinical symptoms, while the decompensated stage is characterized by portal hypertension and severe liver function impairment. Patients are often life‐threatened by complications such as ascites, gastrointestinal bleeding, and hepatic encephalopathy [6]. Complications of cirrhosis usually have an acute onset, critical condition, and high fatality rate. Patients often experience related psychological stress reactions after resuscitation, such as anxiety, depression, and fear. Meanwhile, stress reactions like tension and anxiety can induce sympathetic nervous system excitation, increase heart rate and blood pressure, and may also induce cardiovascular and cerebrovascular diseases, increasing the risk of further bleeding, thereby affecting disease recovery and prognosis [7]. Social support refers to the material and spiritual help an individual obtains through social relationships when coping with stress and handling tense events [8]. Research indicates that social support helps alleviate disease‐related stress symptoms, and a lack of social support is an effective predictor of the severity of post‐traumatic mental health problems [9]. Among various coping strategies, avoidant coping strategies refer to strategies such as denial and withdrawal that mentally or physically avoid stressors to reduce pressure and distract from threats. Studies have found that avoidant coping strategies can provide some immediate benefits for individuals experiencing trauma in the short term but may adversely affect long‐term individual development [10, 11]. Social support helps mobilize resources for individuals to confront trauma directly, forming positive coping [12], thus potentially reducing the use of avoidant coping strategies and subsequently reducing patients' negative psychological problems. Sense of control refers to an individual's belief that they can and do master, control, and shape their own lives [13]. It is a cognitive resource influenced by social support [14]. Research has found that a sense of control is associated with an individual's adaptability to stress [15]. Facing adversity, enhancing the sense of control can reduce an individual's passivity and helplessness, thereby alleviating psychological distress [16], whereas a lower sense of control has been associated with post‐traumatic stress symptoms [17]. The cognitive theory of post‐traumatic stress disorder posits that post‐traumatic distress, intolerance of distress, and avoidance are maintained by irrational beliefs related to self‐control ability or uncontrollable evaluations of trauma‐related emotions and memories [18]. Therefore, a sense of control may be an important psychological resource in patients' adaptation to illness‐related stress. Latent Class Analysis (LCA) is a probability model‐based method for classifying research subjects. It can distinguish homogeneous individuals with superficially similar characteristics into different categories with distinct features, maximizing differences between classes and minimizing differences within classes [19]. In recent years, LCA has been used in symptom classification studies for patients with breast cancer [20], stroke [21], ICU [22], coronary heart disease [23], chronic diseases [24], etc., spanning disciplines such as psychology, medicine, education, mathematics, and economics. Given the impact of posttraumatic stress symptoms, social support, sense of control, and avoidant coping strategies on the quality of life and disease recovery of patients with liver cirrhosis, this study uses latent class analysis to identify patterns across these variables and examines factors associated with class membership, aiming to provide a targeted basis for healthcare professionals in the disease management of patients with liver cirrhosis.

## 2 Materials and Methods

### 2.1 Participants

A convenience sampling method was used to recruit patients with cirrhosis from the outpatient and inpatient services of the hepatology unit at a public tertiary hospital in Shenzhen, Guangdong Province, from October 2024 to May 2025. Inclusion criteria were: a diagnosis of compensated or decompensated cirrhosis; ability to complete the questionnaire independently or with researcher assistance; and provision of informed consent and willingness to participate. Participants aged < 18 years were eligible under the ethics‐approved protocol only when written informed consent was obtained from a legal guardian. Exclusion criteria were other severe chronic diseases (e.g., heart, lung, or renal failure) and cognitive impairment or other mental illness that would prevent questionnaire completion. To support the conceptual interpretation of PTSS in relation to cirrhosis, participants were asked to consider severe cirrhosis‐related experiences, such as the initial diagnosis, a first episode of decompensated complications (e.g., variceal bleeding or ascites), or intensive care unit admission. Because a formal trauma‐screening instrument was not administered, attribution of PTSS specifically to cirrhosis‐related events was interpreted cautiously.

### 2.2 Data Collection

Data were collected using online questionnaires through Wenjuanxing (https://www.wjx.cn) and paper‐based questionnaires. All researchers received standardized training before data collection. The study purpose, procedures, voluntary nature of participation, and confidentiality protections were explained before questionnaire completion. Consent was documented before participation: the online questionnaire required participants to select the informed‐consent option, whereas the paper version used a signed consent form. For the five participants aged < 18 years, written informed consent was obtained from a legal guardian before questionnaire completion, in accordance with the approved protocol. Questionnaires were completed individually and checked for completeness. Completion required approximately 10 min. Questionnaires with incomplete responses or excessive repetitive response patterns were excluded.

### 2.3 Study Tools

#### 2.3.1 General Information Questionnaire

A comprehensive literature review was conducted on personal demographic and clinical variables included in studies of patients with liver cirrhosis. After literature consolidation, the following information was included in this study: age, occupation, gender, education level, marital status, type of medical insurance, income, and cirrhosis stage.

#### 2.3.2 Perceived Social Support Scale (PSSS)

The PSSS was developed by Zimet et al. [25] and systematically revised into the Chinese version by Jiang Qianjin et al. [26]. This scale includes three dimensions: family support, friend support, and significant other support, measuring the degree of support individuals perceive from various social sources such as family, friends, and others, while the total score reflects the overall level of perceived social support. The scale consists of 12 items using a Likert 7‐point scale. Higher total scores indicate higher perceived social support. In this study, the Cronbach's α coefficient for the total scale was 0.950, and for the three dimensions of family support, friend support, and significant other support, the coefficients were 0.867, 0.867, and 0.866, respectively.

#### 2.3.3 Post‐Traumatic Stress Checklist‐Civilian Version (PCL‐C)

The PCL‐C was developed by the US National Center for PTSD and translated into Chinese by Jiang Chao and colleagues [27]. It is used to assess posttraumatic stress symptoms following stressful or traumatic experiences. The 17‐item scale reflects three DSM‐IV symptom clusters: re‐experiencing, avoidance/numbing, and hyperarousal, and uses a 5‐point Likert response format. Higher total scores indicate more severe PTSS. In the Chinese application cited for this study, scores of 17–37, 38–49, and 50–85 were used to describe increasing levels of PTSS [28]. In the present sample, Cronbach's α was 0.955 for the total scale and 0.862, 0.906, and 0.848 for re‐experiencing, avoidance/numbing, and hyperarousal, respectively.

#### 2.3.4 Spheres of Control Scale (SCS)

Developed by Paulhus et al. in 1983, this scale measures an individual's perceived control over their own behavior, interpersonal relationships, and socio‐political aspects [29]. The scale consists of 30 items, divided into three subscales: personal efficacy, interpersonal control, and sociopolitical control. This study used the personal efficacy subscale, which contains 10 items using a Likert 7‐point scale. Higher total scores indicate a stronger sense of control over personal plans, achievements, etc. In this study, the Cronbach's α coefficient for the personal efficacy subscale was 0.924.

#### 2.3.5 Self‐Report Coping Scale (SRCS)

The version revised by Kochenderfer–Ladd and Skinner [30], based on the scale developed by Causey and Dubow in 1992, was used. The scale consists of 22 items, divided into five dimensions: seeking help, problem‐solving, internalizing, externalizing, and withdrawal. The scale uses a Likert 5‐point scale. Higher scores on each dimension indicate greater use of that coping strategy. This study was only interested in avoidant coping strategies. Among these, internalizing, externalizing, and withdrawal belong to avoidant coping strategies. In this study, the overall Cronbach's α coefficient for these three dimensions was 0.839, and for the internalizing, externalizing, and withdrawal dimensions, the coefficients were 0.910, 0.891, and 0.895, respectively.

### 2.4 Ethical Considerations

This study was approved by the ethics committee of Beijing University of Chinese Medicine Shenzhen Hospital (Longgang) (Approval No: KY‐2024‐239‐01). The ethics approval covered the inclusion of participants aged < 18 years. Adult participants provided informed consent before participation. For all five participants aged < 18 years, written informed consent was obtained from a legal guardian before questionnaire completion. For online questionnaires, consent was documented by selecting the informed‐consent option after reviewing the study information; paper questionnaires used a signed consent form. Participation was voluntary, and participants could withdraw without consequences. Questionnaires were completed anonymously, and study data were used only for research purposes and handled confidentially.

### 2.5 Statistical Analysis

After questionnaire collection, data were entered into Excel and analyzed using SPSS version 29.0 and Mplus version 8.7. Normality was assessed using the Shapiro–Wilk test. Normally distributed continuous variables are presented as mean ± standard deviation and were compared using *t*‐tests or analysis of variance, as appropriate. Non‐normally distributed continuous variables are presented as median (P25, P75) and were compared using rank‐sum tests. Categorical variables are presented as frequencies and percentages and were compared using *χ* ^2^ or Fisher's exact tests. Latent class models were estimated in Mplus. Model evaluation considered the log‐likelihood (LL), Akaike information criterion (AIC), Bayesian information criterion (BIC), sample‐size‐adjusted BIC (aBIC), Lo–Mendell–Rubin adjusted likelihood‐ratio test (LMR), bootstrap likelihood‐ratio test (BLRT), entropy, class size, and substantive interpretability. Lower AIC, BIC, and aBIC values indicate better relative fit. Significant LMR or BLRT results support the k‐class model over the (k − 1)‐class model. Entropy ranges from 0 to 1, with higher values indicating clearer classification. All tests were two‐sided with *α* = 0.05.

Regarding sample size, although no formal a priori power analysis was conducted due to the exploratory nature of the study, a post‐hoc sensitivity analysis was performed using G*Power 3.1 to evaluate the statistical power of the multivariate logistic regression models. For the most complex model (with 5 predictors, alpha = 0.05, and a medium effect size of OR = 1.5), the achieved power was 0.87, which exceeds the conventional 0.80 threshold, suggesting that the sample size (*n* = 316) was sufficient to detect meaningful associations. Additionally, methodological literature recommends a minimum sample size of 300 for stable latent class estimation, and our sample meets this benchmark.

## 3 Results

### 3.1 Common Method Bias

To assess potential artificial covariance between predictor and criterion variables caused by the same data source, measurement environment, item context, and item characteristics, this study used Harman's single‐factor test to evaluate common method bias. SPSS 29.0 was used to perform unrotated exploratory factor analysis on all items from the Perceived Social Support Scale, PCL‐C, Spheres of Control Scale, and Self‐Report Coping Scale. The analysis revealed 5 factors with eigenvalues greater than 1. The first factor accounted for 25.49% of the variance, below the critical threshold of 50% [31]. Therefore, no significant common method bias was present in this study.

### 3.2 Participant General Information

We invited 326 patients to participate. Questionnaires from 10 patients were excluded as invalid, leaving 316 participants for analysis (valid‐response rate, 96.93%). Demographic and clinical characteristics are shown in Table 3. Because latent class recovery depends on class separation, indicator characteristics, sample size, and model complexity rather than a single universal sample‐size rule [32, 33], the adequacy of the solution was evaluated using multiple fit indices, entropy, class size, and substantive interpretability. Particular caution was applied to Class 2 because it contained only 24 participants.

### 3.3 Levels of Social Support, Post‐Traumatic Stress Symptoms, Sense of Control, and Avoidant Coping Strategies in Patients With Liver Cirrhosis

In this survey, the social support score for patients with liver cirrhosis was 60.78 ± 16.00; the post‐traumatic stress symptom score was 30.67 ± 11.99, with 53 patients (16.7%) having relatively obvious post‐traumatic stress symptoms; the total score for sense of control was 48.42 ± 12.07; and the score for avoidant coping strategies was 29.53 ± 9.26. Patients with compensated and decompensated cirrhosis exhibit significant differences in social support and avoidance behaviors. Detailed data are shown in Table 1.

**Table 1:** Stage‐stratified descriptive statistics of the four psychological variables.

	Compensated stage ($\bar{x}$ ± *S*)	Decompensated stage ($\bar{x}$ ± *S*)	*T*	*p*
Perceived social support	63.02 ± 13.797	59.27 ± 17.216	2.055	0.041
Posttraumatic stress symptoms	29.84 ± 10.377	31.23 ± 12.976	−1.008	0.314
Sense of control	49.82 ± 12.107	47.46 ± 11.987	1.709	0.088
Avoidant coping strategies	26.01 ± 7.137	31.93 ± 9.791	−5.859	0.001

The PCL‐C threshold of 38 points was retained as the study's operational threshold for elevated PTSS based on the cited Chinese application [28]. Published PCL‐C studies have used different cut points depending on population and screening purpose [28, 34]; therefore, this threshold should not be interpreted as a diagnostic cutoff. A score of 38 corresponds to 47.7% of the PCL‐C maximum score. Because validated clinical cutoffs were not available for the PSSS, SCS, and avoidant‐coping indicators in this cirrhosis population, study‐specific operational thresholds were created using the same proportion of each scale's possible score: PSSS ≥ 38 was coded as social support present; SCS ≤ 32 as low sense of control and ≥ 33 as preserved sense of control; and avoidant‐coping scores ≥ 34 as higher avoidant coping. These dichotomizations were used only for the latent class indicators and are not clinical diagnostic thresholds.

### 3.4 Latent Classes of Social Support, Post‐Traumatic Stress Symptoms, Sense of Control, and Avoidant Coping Strategies

#### 3.4.1 Results of Latent Class Analysis

Latent class models with one through four classes were estimated. The information criteria were lowest for the two‐class model, whereas the LMR and BLRT remained significant for the three‐class model and were not significant for the four‐class model. Considering statistical fit, classification quality, class size, and substantive interpretability together, the three‐class solution was selected (Table 2). Its entropy was 0.984, indicating clear classification, and both the LMR (*p* = 0.033) and BLRT (*p* = 0.037) favored three classes over two. The four‐class model did not provide a significant improvement over the three‐class model.

**Table 2:** The latent class model result (*n* = 316).

Model	LL	AIC	BIC	a BIC	Entropy	*p*		Class probability
						LMR	BLRT	
1	−517.283	1042.566	1057.588	1044.902	—	—	—	1.00
2	−454.915	927.831	961.633	933.087	0.998	< 0.001	< 0.001	0.076/0.924
3	−451.245	930.490	983.071	938.666	0.984	0.033	0.037	0.642/0.075/0.282
4	−451.114	940.227	1011.586	951.232	0.911	0.641	0.643	0.056/0.107/0.783/0.054

Abbreviations: AIC, Akaike information criterion; aBIC, sample‐size‐adjusted BIC; BIC, Bayesian information criterion; BLRT, bootstrap likelihood‐ratio test; LL, log‐likelihood; LMR, Lo‐Mendell‐Rubin adjusted likelihood‐ratio test.

#### 3.4.2 Determination and Naming of Latent Classes

Class 1 included 203 participants (64.2%) and was characterized by social support, a high probability of no clinically elevated PTSS, preserved sense of control, and a lower tendency toward avoidant coping; it was therefore labeled the “Buffering Type—Low Avoidance/Symptoms Group.” Class 2 included 24 participants (7.5%) and was characterized by low social support and low sense of control, together with a mixed PTSS/avoidance profile; it was labeled the “Buffering Deficiency—Symptoms/Avoidance Dilemma Group.” Class 3 included 89 participants (28.2%) and was characterized by low social support, elevated PTSS, lower sense of control, and frequent avoidant coping; it was labeled the “Avoidant Coping—Symptoms Dilemma Group.” Because Class 2 was small, its profile and subsequent class‐specific association estimates should be regarded as provisional pending replication. Conditional response probabilities for the three classes are shown in Figure 1.

**Figure 1:**
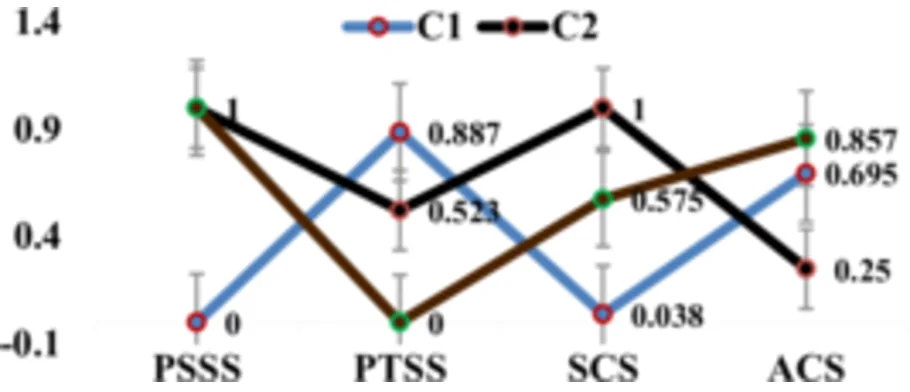
Conditional response probabilities for the three‐class model of social support, PTSS, sense of control, and avoidant coping strategies in patients with cirrhosis.

### 3.5 Analysis of Influencing Factors for Latent Classes

Univariate analyses showed that latent class distributions differed by occupational status, gender, marital status, monthly income, and cirrhosis stage (all *p* < 0.05; Table 3). The class distribution also differed markedly by disease stage: among 128 patients with compensated cirrhosis, 113 (88.3%) were in Class 1, 5 (3.9%) in Class 2, and 10 (7.8%) in Class 3; among 188 patients with decompensated cirrhosis, 90 (47.9%) were in Class 1, 19 (10.1%) in Class 2, and 79 (42.0%) in Class 3 (Pearson *χ* ^2 ^= 54.852, df = 2, *p* < 0.001; Table 4). Variables significant in univariate analyses were entered into the multinomial logistic regression model (assignments in Table 5). In the pooled adjusted model, decompensated stage differentiated Class 2 from Class 1 (*p *= 0.003). Unmarried status, monthly income < 5000 RMB, unemployment, and decompensated stage differentiated Class 3 from Class 1 (all *p* ≤ 0.001). Gender was not significant in the adjusted C1‐versus‐C3 comparison after correction of the reported test statistic (OR = 0.780, 95% BCa bootstrap CI 0.365–1.667, *p* = 0.523). Marital and occupational status also differentiated Class 3 from Class 2. Detailed estimates are shown in Table 6.

**Table 3:** Demographic characteristics of survey subjects and the results of univariate analysis of their three latent classes (*n* = 316 (%)).

Characteristic	Group	Frequency (%)	C1 (*n* = 203)	C2 (*n* = 24)	C3 (*n* = 89)	*χ* ^2^	*p*
Occupation	Employed	238 (75.3)	185 (91.1)	20 (83.3)	33 (37.1)	98.143	< 0.001
	Unemployed	78 (24.7)	18 (8.9)	4 (16.7)	56 (62.9)		
Gender	Male	212 (67.1)	123 (60.6)	14 (58.3)	75 (84.3)	16.61	< 0.001
	Female	104 (32.9)	80 (39.4)	10 (41.7)	14 (15.7)		
Marriage	Married	234 (74.1)	171 (84.2)	20 (83.3)	43 (48.3)	42.714	< 0.001
	Unmarried	82 (25.9)	32 (15.8)	4 (16.7)	46 (51.7)		
Age	< 18 Years	5 (1.6)	4 (80.0)	1 (20.0)	0 (0.0)	4.771	0.452
	18–30 Years old	43 (13.6)	28 (65.2)	4 (9.3)	11 (25.6)		
	30–40 Years old	94 (29.7)	59 (62.8)	8 (8.5)	27 (28.7)		
	40–50 Years old	111 (35.1)	73 (65.8)	8 (7.2)	30 (27.0)		
	> 50 Years old	63 (19.9)	39 (61.9)	3 (4.8)	21 (33.3)		
Education	Junior college or below	115 (36.4)	72 (62.6)	5 (4.3)	38 (33.0)	5.745	0.204
	Undergraduate	178 (56.3)	113 (63.6)	18 (10.1)	47 (26.4)		
	Master's degree or above	23 (7.3)	18 (78.3)	1 (4.3)	4 (17.4)		
Medical insurance	Yes	257 (81.3)	167 (65.0)	16 (6.2)	74 (28.8)	3.709	0.160
	No	59 (18.7)	36 (61.0)	8 (13.6)	15 (25.4)		
Income	< 5000 RMB	130 (41.1)	57 (28.1)	10 (41.7)	63 (70.8)	46.608	< 0.001
	≥ 5000 RMB	186 (58.9)	146 (71.9)	14 (58.3)	26 (29.2)		
Disease stage	Compensated stage	128 (40.5)	113 (55.7)	5 (20.8)	10 (11.2)	54.85	< 0.001
	Decompensated stage	188 (59.5)	90 (44.3)	19 (79.2)	79 (88.8)		

*Note:* C1, buffering type—low avoidance/symptoms group; C2, buffering deficiency—symptoms/avoidance dilemma group; C3, avoidant coping—symptoms dilemma group.

**Table 4:** Distribution of latent class membership according to cirrhosis stage.

	Group	Frequency (%)	C1 (*n* = 203)	C2 (*n* = 24)	C3 (*n* = 89)	*χ* ^2^	*p*
Disease stage	Compensated stage	128 (40.5)	113 (88.3)	5 (3.9)	10 (7.8)	54.85	< 0.001
	Decompensated stage	188 (59.5)	90 (47.9)	19 (10.1)	79 (42.0)		

**Table 5:** Assignment of independent variables.

Variable	Value
Occupation	Employed = 1; unemployed = 2
Gender	Male = 1; female = 2
Marriage	Married = 1; unmarried = 2
Income	< 5000 RMB = 1; ≥ 5000 RMB = 2
Disease stage	Compensated stage = 1; decompensated stage = 2

**Table 6:** Multinomial logistic regression analysis of three latent classes.

	Option	*β*	Standard error	Wald	df	*p*	OR	95% BCa bootstrap CI	
								Lower limit	Upper limit
C1 compared to C2	Intercept	−0.822	0.899	0.836	1	0.360	—	—	—
	Marriage	0.074	0.609	0.015	1	0.903	1.077	0.326	3.551
	Income	0.567	0.471	1.454	1	0.228	1.764	0.701	4.436
	Occupation	−0.756	0.635	1.418	1	0.234	0.470	0.124	1.662
	Gender	−0.496	0.455	1.184	1	0.276	0.609	0.249	1.487
	Disease stage	−1.589	0.526	9.117	1	0.003	0.204	0.073	0.573
C1 compared to C3	Intercept	2.470	0.608	16.486	1	< 0.001	—	—	—
	Marriage	−1.530	0.393	15.180	1	< 0.001	0.217	0.100	0.468
	Income	1.211	0.372	10.583	1	0.001	3.356	1.618	6.959
	Occupation	−2.799	0.424	43.578	1	< 0.001	0.061	0.025	0.143
	Gender	−0.248	0.388	0.409	1	0.523	0.780	0.365	1.667
	Disease stage	−2.707	0.494	30.036	1	< 0.001	0.067	0.025	0.176
C3 compared to C2	Intercept	−3.292	0.899	13.403	1	< 0.001	—	—	—
	Marriage	1.604	0.624	6.602	1	0.010	4.972	1.463	16.896
	Income	−0.643	0.524	1.509	1	0.219	0.526	0.188	1.467
	Occupation	2.043	0.616	10.984	1	0.001	7.713	2.023	24.575
	Gender	−0.247	0.518	0.228	1	0.633	0.781	0.283	2.155
	Disease stage	1.118	0.674	2.749	1	0.097	3.060	0.816	11.477

*Note:* Likelihood‐ratio *χ* ^2 ^= 182.613, *p* < 0.001. Goodness‐of‐fit test *p* values were > 0.05, and the maximum pseudo‐R^2^ was 0.439. Bootstrap CIs were based on 1000 bias‐corrected and accelerated (BCa) resamples.

To evaluate the precision of regression estimates, particularly those involving the small Class 2 (*n *= 24), bias‐corrected and accelerated (BCa) bootstrap 95% confidence intervals were generated from 1000 resamples (Table 6). Several intervals involving Class 2 were wide, reflecting substantial uncertainty, so these estimates should not be treated as stable class‐specific effects. A sensitivity analysis excluding Class 2 and re‐estimating the Class 1 versus Class 3 model produced the same direction and statistical‐significance pattern for the remaining predictors. This supports the C1‐versus‐C3 findings but does not remove the uncertainty surrounding the existence, prevalence, or predictors of Class 2.

Stage‐stratified multinomial logistic regression analyses were performed with Class 1 as the reference outcome and simultaneous adjustment for marital status, monthly income, occupational status, and gender (Table 7). In the compensated subgroup, unmarried status was associated with higher odds of Class 3 rather than Class 1 membership (OR = 34.04, 95% CI 3.54–327.55, *p* = 0.002). No other predictor reached statistical significance for Class 3 versus Class 1, although unemployment showed a borderline association (OR = 5.20, 95% CI 0.96–28.01, *p* = 0.055), and no predictor was significantly associated with Class 2 versus Class 1. Because only five compensated patients were assigned to Class 2 and 10 to Class 3, these estimates were imprecise and should be interpreted cautiously.

**Table 7:** Stage‐stratified multinomial logistic regression of factors associated with latent class membership.

Disease stage	Predictor	Class 2 vs. class 1 OR (95% CI)	*p*	Class 3 vs. class 1 OR (95% CI)	*p*
Compensated (*n* = 128)	Unmarried vs. married	3.62 (0.48–27.51)	0.214	34.04 (3.54–327.55)	**0.002**
	Income < 5000 vs. ≥ 5000 RMB	0.95 (0.13–6.89)	0.963	1.02 (0.18–5.79)	0.984
	Unemployed vs. employed	1.35 (0.13–14.39)	0.804	5.20 (0.96–28.01)	0.055
	Female vs. male	0.33 (0.03–3.14)	0.336	0.20 (0.02–2.13)	0.183
Decompensated (*n* = 188)	Unmarried vs. married	0.39 (0.08–1.96)	0.256	2.87 (1.18–6.95)	0.020
	Income < 5000 vs. ≥ 5000 RMB	2.24 (0.76–6.58)	0.143	4.45 (1.93–10.29)	< 0.001
	Unemployed vs. employed	3.00 (0.62–14.48)	0.170	21.79 (7.51–63.21)	< 0.001
	Female vs. male	2.22 (0.79–6.22)	0.128	0.58 (0.22–1.52)	0.267

In the decompensated subgroup, unmarried status (OR = 2.87, 95% CI 1.18–6.95, *p* = 0.020), monthly income < 5000 RMB (OR = 4.45, 95% CI 1.93–10.29, *p* < 0.001), and unemployment (OR = 21.79, 95% CI 7.51–63.21, *p* < 0.001) were associated with higher odds of Class 3 versus Class 1 membership after adjustment. Gender was not significantly associated with Class 3 membership in this subgroup, and none of the four demographic factors was significantly associated with Class 2 versus Class 1.

## 4 Discussion

### 4.1 Posttraumatic Stress Symptoms, Buffering Resources, and Avoidant Coping Strategies in Patients With Cirrhosis

This study identified heterogeneity in the joint patterning of perceived social support, PTSS, sense of control, and avoidant coping among patients with cirrhosis. Overall mean scores showed substantial between‐person variation, and 16.7% of participants met the study's operational threshold for elevated PTSS. This value describes the proportion of the present cross‐sectional sample meeting that threshold and should not be interpreted as an incidence estimate or a diagnosis of PTSD.

The three‐class solution provides a descriptive framework for the co‐occurrence of psychosocial resources, PTSS, and avoidant coping. Class 1, the Buffering Type—Low Avoidance/Symptoms Group (64.2%), was characterized by greater perceived social support and sense of control, together with fewer PTSS and less avoidant coping. This pattern is compatible with prior work linking social support and perceived control with better psychological adaptation in chronic illness, although the present cross‐sectional analysis cannot establish a buffering mechanism or direction of effect [35, 36, 37, 38, 39]. Class 2, the Buffering Deficiency— Symptoms/Avoidance Dilemma Group, represented only 24 participants (7.5%). Its profile of low social support and low sense of control with mixed PTSS and avoidance may indicate limited psychosocial resources, but this interpretation is tentative. The small class size and wide class‐specific regression confidence intervals mean that its distinctiveness, prevalence, and associated factors require replication in larger samples; the present data do not justify a definitive vulnerability ranking among classes [32, 33]. Class 3, the Avoidant Coping —Symptoms Dilemma Group (28.2%), showed low social support, elevated PTSS, lower sense of control, and frequent avoidant coping. This co‐occurrence is consistent with meta‐analytic and theoretical literature showing an association between avoidance‐oriented coping and greater posttraumatic distress [40, 41]. However, because PTSS and coping were measured concurrently, the present findings cannot determine whether avoidance preceded PTSS, resulted from PTSS, or reflected a reciprocal process. Longitudinal studies are needed to test whether social support and sense of control prospectively buffer symptom development or whether symptom burden itself erodes perceived resources.

### 4.2 Analysis of Influencing Factors for Latent Classes

Class membership also varied according to several demographic and clinical characteristics. In univariate analyses, occupational status, gender, marital status, monthly income, and disease stage were associated with class distribution. These findings indicate statistical associations, not evidence that these characteristics cause a particular coping style or psychological profile.

In the pooled multivariable analysis, unmarried status, monthly income < 5000 RMB, unemployment, and decompensated cirrhosis differentiated Class 3 from Class 1. These associations are broadly compatible with evidence that social and functional vulnerability can accompany poorer patient‐reported outcomes in cirrhosis and other chronic diseases [39, 42]. Importantly, gender should not be interpreted as an independent predictor in this study. Although class distribution differed by gender in the univariate analysis, the corrected adjusted C1‐versus‐C3 estimate was not statistically significant (OR = 0.780, 95% BCa bootstrap CI 0.365–1.667, *p* = 0.523), and gender was also non‐significant in both stage‐stratified models. The unadjusted gender difference may therefore reflect confounding or sampling variability and requires replication rather than a biological or sociocultural explanation from the present data.

Disease stage showed the clearest and most consistent clinical association with class membership. Patients with decompensated cirrhosis were more frequently classified in Classes 2 and 3 than patients with compensated disease. This is clinically plausible because decompensation is accompanied by complications such as ascites, variceal bleeding, and hepatic encephalopathy, recurrent healthcare use, impaired functioning, and lower health‐related quality of life [4, 6, 43, 44]. In the adjusted model, disease stage remained statistically associated with class membership after accounting for the included demographic factors. This should be interpreted as an independent statistical association rather than evidence that decompensation causes the observed psychological profile. Nonetheless, the marked difference in class distribution supports consideration of psychological assessment when patients experience decompensation, particularly because patient‐reported mental health, social function, and symptom burden are important dimensions of cirrhosis care [45].

### 4.3 Disease Stage Stratification Analysis

The stage‐stratified analyses further illustrate heterogeneity that was less visible in the pooled model. Class 3 accounted for 7.8% of patients with compensated cirrhosis and 42.0% of those with decompensated cirrhosis, whereas Class 1 decreased from 88.3% to 47.9%. Because the study was cross‐sectional, these between‐group differences do not demonstrate that individual patients transition from one latent class to another as cirrhosis progresses. They do, however, suggest that the period around clinical decompensation may be a useful point for more intensive psychological assessment.

Within the decompensated subgroup, unmarried status, monthly income below 5000 RMB, and unemployment remained associated with Class 3 rather than Class 1 membership after adjustment, suggesting that social and economic vulnerability may further distinguish psychological profiles even within the same broad disease stage. In the compensated subgroup, unmarried status was also associated with Class 3 membership; however, the confidence interval was very wide because only five compensated participants were in Class 2, and 10 were in Class 3. These subgroup estimates should therefore be considered exploratory.

Taken together, the findings support a risk‐stratified assessment approach rather than immediate class‐specific treatment recommendations. Psychological screening may be considered throughout the cirrhosis trajectory and intensified when decompensation occurs, with attention to patients who also have limited social or economic resources. Prospective studies are needed to determine whether these latent profiles are reproducible, stable over time, and useful for selecting or tailoring interventions.

## 5 Limitations

This study has several limitations. First, it was conducted at a single‐center with a moderate total sample, which may limit generalizability to other regions, healthcare settings, and patient populations. Second, the study did not systematically assess the specific traumatic events experienced by each participant. Although cirrhosis‐related severe events were defined as the relevant stress context, some PTSS may have reflected earlier, or unrelated trauma, and different cirrhosis‐related events may have different psychological effects. Third, continuous scale scores were dichotomized using study‐defined operational thresholds. This can result in information loss, reduced statistical power, and class solutions that depend on the selected cut points; future work should consider latent profile models using continuous indicators and should validate clinically meaningful thresholds where available. Fourth, Class 2 contained only 24 participants (7.5%). The BCa bootstrap analysis quantified some of the uncertainty but cannot fully resolve the instability caused by a small latent class. Accordingly, the Class 2 profile and its predictor estimates are provisional and require external replication in larger, preferably multicenter samples [32, 33]. Fifth, the cross‐sectional design precludes conclusions about temporal ordering or causality among disease stage, psychosocial resources, coping, and PTSS.

## 6 Conclusion

This study identified three latent classes of patients with cirrhosis based on PTSS, perceived social support, sense of control, and avoidant coping. Class 1 (64.2%) showed a profile of stronger buffering resources with fewer symptoms and less avoidance. Class 2 (7.5%) showed low social support and sense of control with a mixed PTSS/avoidance profile; because this class contained only 24 participants, it should be regarded as a provisional subgroup requiring replication. Class 3 (28.2%) showed low social support, elevated PTSS, lower sense of control, and frequent avoidant coping.

In adjusted analyses, decompensated cirrhosis differentiated Class 2 from Class 1, while unmarried status, lower monthly income, unemployment, and decompensated cirrhosis differentiated Class 3 from Class 1. Gender was associated with class distribution in univariate analysis but was not statistically significant in the corrected adjusted C1‐versus‐C3 comparison or in the stage‐stratified models.

These findings are associative rather than causal and primarily support further risk stratification and hypothesis generation. Disease stage and social or economic vulnerability may help identify patients who warrant closer psychosocial assessment, but the latent classes, especially the small Class 2, should be validated prospectively before they are used to guide class‐specific interventions.

## Author Contributions

**Jintao Cheng:** conceptualization, writing – review and editing, formal analysis, software, resources, visualization. **Huiqiong Gui:** conceptualization, funding acquisition, supervision, visualization. **Suyun Guo:** conceptualization, validation, resources, project administration, writing – review and editing. **Yihua Yang:** conceptualization, writing – review and editing, formal analysis, software, resources, visualization. **Yun Ran:** conceptualization, resources, data curation, writing – review and editing.

## Ethics Statement

This study was approved by the Ethics Committee of Beijing University of Chinese Medicine Shenzhen Hospital (Longgang) (Approval No.: KY‐2024‐239‐01) and was conducted in accordance with applicable institutional and ethical requirements. Adult participants provided informed consent before participation. For the five participants aged < 18 years, the ethics approval included their participation, and written informed consent was obtained from a legal guardian before questionnaire completion.

## Conflicts of Interest

The authors declare no conflicts of interest.

## Transparency Statement

The corresponding author, on behalf of all authors, confirms that this manuscript is an honest, accurate, and transparent account of the study being reported; that no important aspects of the study have been omitted; and that any discrepancies from the study as planned (and, if relevant, registered) have been explained. This study was not pre‑registered. Therefore, there are no discrepancies from a registered protocol to report.

## Data Availability

The research data are not shared.
